# Telehealth for Assessing and Managing Tardive Dyskinesia: Expert Insights from a Cross-Disciplinary Virtual Treatment Panel

**DOI:** 10.1089/tmj.2022.0234

**Published:** 2023-07-04

**Authors:** Rif S. El-Mallakh, Amy Belnap, Sanjay Iyer, Jeremy Schreiber, Desiree Matthews, Linda Lefler, Daniel Dees, Allen Bott, Nora Vanegas-Arroyave, Adam Wolff, Ulises Pesce, Khody Farahmand, Chirag Shah, Leslie Lundt

**Affiliations:** ^1^ Department of Psychiatry and Behavioral Sciences, University of Louisville School of Medicine, Louisville, Kentucky, USA.; ^2^ Rocky Mountain Psychiatry, Pocatello, Idaho, USA.; ^3^ Neurocrine Biosciences, Inc., San Diego, California, USA.; ^4^ Memory & Movement Charlotte, Charlotte, North Carolina, USA.; ^5^ West Liberty University, West Liberty, West Virginia, USA.; ^6^ Monarch, Charlotte, North Carolina, USA.; ^7^ St. Anthony's Hospital and Bayfront Health St. Petersburg, St. Petersburg, Florida, USA.; ^8^ Department of Neurology, University of South Alabama College of Medicine, Mobile, Alabama, USA.; ^9^ Oakland, California, USA.; ^10^ Baylor College of Medicine—Neurology, Houston, Texas, USA.; ^11^ Denver Neurological Clinic, Denver, Colorado, USA.; ^12^ Department of Psychiatry, University of South Dakota, Vermillion, South Dakota, USA.

**Keywords:** assessment, management, tardive dyskinesia, TD, telehealth, virtual visit

## Abstract

**Introduction:**

Publications on the integration of telehealth in the care of patients with movement disorders are increasing, but little has been presented regarding its use in tardive dyskinesia (TD), a drug-induced movement disorder associated with prolonged exposure to dopamine receptor blocking agents. This study was conducted to address that knowledge gap, based on insights from a panel of TD experts.

**Methods:**

In 2020, six neurologists, three psychiatrists, and three psychiatric nurse practitioners participated in individual semistructured interviews about in-person and virtual TD assessment and management in their practices. Two virtual roundtables were then conducted to consolidate findings from these interviews.

**Results:**

The panel agreed that despite the challenges of virtual TD assessment (e.g., technology issues, difficulty observing entire body, inability to conduct thorough neurological examinations), telehealth can offer benefits (e.g., fewer missed appointments, reduced time/cost, easier access to family/caregiver feedback). The panel also agreed that telehealth should be combined with periodic in-person visits, and they recommended an in-person TD assessment within 6 months before the first virtual visit and at least one in-person assessment every 6 months thereafter. Additional best practices for TD telehealth included implementing video, involving family/caregivers, and providing preappointment instructions to help patients prepare their technology and environment.

**Conclusions:**

Telehealth is not a substitute for in-person visits but can be a helpful complement to in-person clinical care. Clinicians can optimize virtual visits in patients at risk of TD by using targeted questions to identify TD and evaluate its impact and by providing education about approved TD treatments.

## Introduction

Telehealth, which typically involves real-time or store-and-forward audio, text messaging, or video communication,^1,2^ was recommended by the Centers for Disease Control and Prevention (CDC) in February 2020 as part of the federal response to the COVID-19 pandemic.^3^ The pandemic-related stay-at-home orders and subsequent infection mitigation measures prompted wide-ranging changes to health care, including relaxed regulations for providing telehealth and temporary reimbursement parities between in-person and telehealth visits.^3–5^ These factors led to a steep decline in in-person care and a rapid rise in telehealth use from March to June 2020.^3–8^ Based on data from a FAIR Health database with 35+ billion health insurance claims, telehealth usage climbed from 0.2% of monthly claims in January 2020 to a peak of 13% in April 2020 before decreasing to 6% by July 2020 and slowly tapering to 4% in November 2021 (https://www.fairhealth.org/states-by-the-numbers/telehealth).

This uptake of telehealth was particularly evident in psychiatry: 81% of respondents to an American Psychiatric Association (APA) member survey indicated that they were continuing to use telehealth for 75–100% of patients as of January 2021.^9^ According to a recent report from Trilliant Health based on a nationally representative health care claims dataset, ∼56 million Americans used telehealth in 2020 and 2021, with behavioral health diagnoses accounting for 58% of telehealth visits in 2021.^10^ In light of recent trends and telehealth policy changes under consideration (as of February 2022) to extend or permanently adopt measures enacted during the COVID-19 pandemic, it is evident that telehealth will remain a part of the health care delivery system.^11–13^

The potential benefits and challenges of telehealth depend on multiple factors, including clinicians' concerns, patients' needs, and reasons for the visit (e.g., initial diagnosis, follow-up assessment, medication adjustments or other treatment-related issues).^14–20^ To that end, a panel of experts was convened to better understand how telehealth may be applied to assessing, diagnosing, and treating patients with tardive dyskinesia (TD), a hyperkinetic movement disorder associated with prolonged exposure to antipsychotics and other dopamine receptor blocking agents, such as metoclopramide. TD can impair a patient's physical, mental, and emotional well-being, leading to feelings of embarrassment, anxiety, and social withdrawal/isolation.^21–25^ Patients' TD can also negatively impact their caregivers, who are often their family or friends.^26^

Despite the availability of approved TD medications (e.g., vesicular monoamine transporter 2 [VMAT2] inhibitors, valbenazine and deutetrabenazine), identification, diagnosis, and assessment of this disorder remains complex, and these complexities can be compounded when care is delivered via telehealth.^22^ Further education regarding appropriate screening, management, and treatment of TD is important, especially for telehealth visits where identification and diagnosis may be more difficult or impossible, as in the case of audio-only telehealth interventions.

In their 2020 treatment guidelines for schizophrenia, the APA recommends that all patients taking an antipsychotic should be screened for TD and other drug-induced movement disorders (DIMDs), with follow-up clinical assessments at all visits (e.g., visual observation) and a formal evaluation every 12 months with a structured instrument such as the Abnormal Involuntary Movement Scale (AIMS).^27^ The APA guidelines also suggest that patients at high risk of developing TD be screened every six months. In a recent modified Delphi consensus study of the screening, diagnosis, and treatment of TD, consensus was reached that brief screening for TD should be performed at every clinical encounter in all patients taking antipsychotics, and that management of TD requires an overall assessment of psychopharmacologic treatment, including use of antipsychotics, anticholinergics, and VMAT2 inhibitors.^28^ Here, we present insights from an expert panel on how to implement these types of recommendations during virtual visits.

## Methods

Twelve health care providers (HCPs) were identified based on their research and/or clinical experience with TD, both in person and via telehealth. These HCPs were invited by the study sponsor (Neurocrine Biosciences, Inc., San Diego, CA) to participate in an expert panel on TD; all 12 individuals accepted the invitation. The cross-disciplinary panel included six neurologists, three psychiatrists, and three psychiatric nurse practitioners with diverse backgrounds, practicing in distinct U.S. regions, and from various types of practice settings involving the management of TD.

This qualitative study was conducted using an iterative approach developed by Metaplan^®^ (Princeton, NJ) to elicit feedback on key topics regarding the multilayered components of TD management (i.e., screening, diagnosis, assessment, treatment).^29,30^ The process began in July 2020 with 60-min online interviews that were conducted by Metaplan with each panelist. The interviews were semistructured, based on a question/discussion guide that was developed by the study sponsor, but representatives of the sponsor did not participate or influence the discussions in any way. Results from the initial interviews were collated using Metaplan's dialogue mapping format and analyzed to determine areas of convergence and divergence regarding the various key topics. These findings, which were distributed to all panelists, were then used to guide a second round of 60-min semistructured interviews conducted by Metaplan.

Results from the second round of interviews were analyzed for convergence/divergence and used to generate two concurrent workstreams that culminated in two workshops facilitated by Metaplan in October 2020 that used visualization (e.g., virtual “sticky notes”) and moderated discussions to identify key areas of consensus. One virtual workshop, which included four neurology specialists over two sessions, focused on the screening and diagnosis of TD.^31^ The other workshop, which included five psychiatry specialists, focused on the assessment and management of TD in telehealth settings. Findings from both workshops were presented and discussed during a final virtual workshop in November 2020 that included 11 of the 12 expert panelists. Results from all interviews and virtual workshops were documented and analyzed by Metaplan. Telehealth-related findings are presented in the current report. No quantitative or statistical methods were applied.

## Results

### INDIVIDUAL INTERVIEWS: TD SCREENING, DIAGNOSIS, AND TREATMENT

**Fig. 1.**
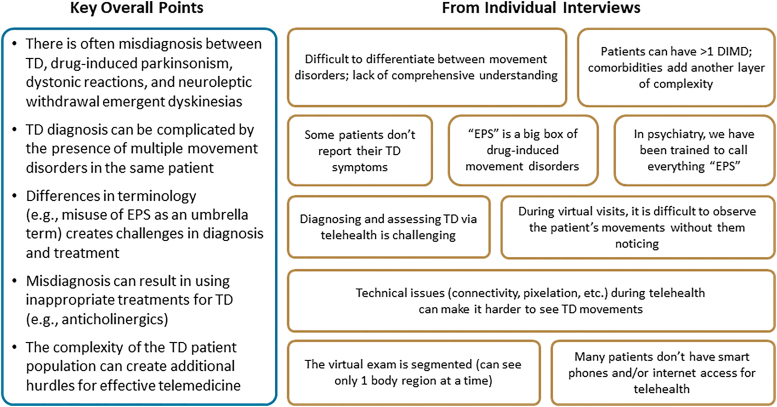
Results from individual interviews: challenges of evaluating TD. DIMD, drug-induced movement disorder; EPS, extrapyramidal symptoms; TD, tardive dyskinesia.

During their individual interviews, experts described some of the inherent challenges of assessing TD in both in-person and virtual settings (*Fig. 1*). Diagnostic challenges included difficulty differentiating TD from other DIMDs and the misuse of “extrapyramidal symptoms” (EPS) as an umbrella term for all DIMDs, which can be detrimental to patient outcomes, as TD is distinct from other DIMDs in phenomenology and treatment. TD identification and differential diagnosis can also be complicated by the presence of multiple movement disorders in the same patient (e.g., TD and parkinsonism), as well as the overlap of symptoms from multiple psychiatric and/or medical comorbidities.

Another challenge to arriving at the correct diagnosis is that some patients may not report their abnormal movements because of a lack of understanding of what caused these movements and how they impact their overall psychiatric or medical well-being, decreased awareness due to cognitive deficits from underlying chronic schizophrenia, or even inconsistency of TD symptoms (i.e., waxing and waning in severity and/or intensity).

In addition to the inherent challenges in the screening, diagnosis, and treatment of TD, telehealth adds another level of complexity. For example, the panelists described the technical challenges of virtual TD assessment, such as the segmented views of a patient's body on video (clinicians cannot diagnose or assess movements which they cannot see). Visualization can also be hindered by pixelation or inadequate lighting. Moreover, during virtual visits, it is difficult for HCPs to observe a patient's full-body movements or posture in an unobtrusive way (i.e., without asking the patient to adjust the camera and/or their position in front of the camera). Some patients try to control or hide their movements when they know they are being observed, which can hinder a clinician's overall assessment.

Finally, the panelists noted that many patients do not have smart phones and/or internet access, or they may have difficulty using this technology. In instances where only audio-only telehealth is provided, it is likely that screening for DIMDs is not possible and/or not conducted at all.

**Fig. 2.**
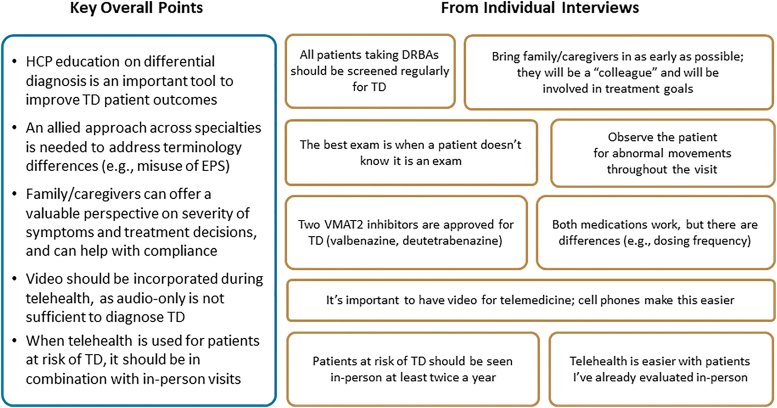
Results from individual interviews: Strategies for improving TD screening, diagnosis, and treatment. DRBA, dopamine receptor blocking agent; EPS, extrapyramidal symptoms; HCP, health care provider; TD, tardive dyskinesia; VMAT2, vesicular monoamine transporter 2.

During their individual interviews, the panelists suggested strategies to improve TD assessment (both in-person and virtual), including HCP education on the phenomenology and differential diagnosis of TD, as this is a key tool for guiding treatment selection and improving patient outcomes (*Fig. 2*). In addition, terminology differences across specialties (e.g., misuse of EPS as a catch-all term) need to be addressed to ensure TD is appropriately diagnosed and treated. Panelists also suggested engaging with family members and caregivers to gather additional information about the presence and impact of abnormal involuntary movements. Family/caregivers can also help patients with treatment decisions and help support adherence to any prescribed medications; thus, clinicians should educate patients and their families about approved TD medications (i.e., valbenazine and deutetrabenazine).

Finally, the experts suggested that when telehealth visits are used for patients who have or are at risk of developing DIMDs, they should be used in combination with periodic in-person visits. Although suboptimal when compared directly to in-person evaluations, video-enabled communication should always be incorporated into virtual visits. Audio-only interactions are completely inadequate for assessing TD.

### ROUNDTABLE WORKSHOPS: TD TELEHEALTH

**Fig. 3.**
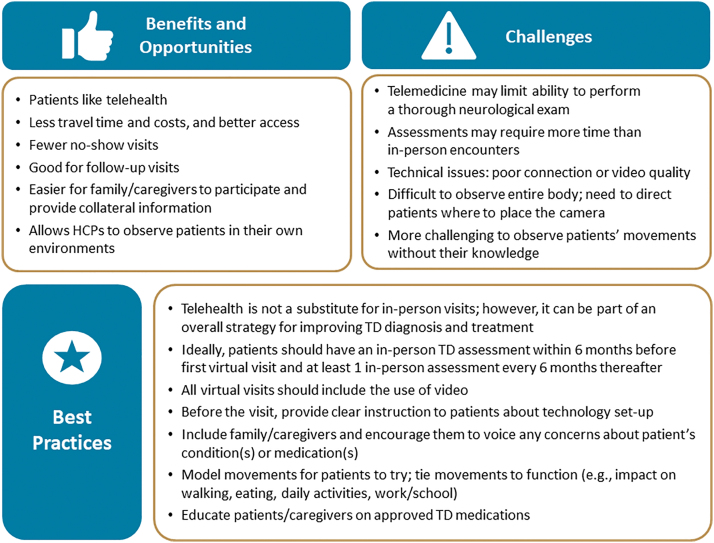
Key panel findings: telehealth for TD. HCP, health care provider; TD, tardive dyskinesia.

After the individual interviews were completed, the expert panel met for two roundtable workshops to discuss the potential benefits, challenges, and best practices for telehealth in the management of TD. Key findings from these workshops are presented in *Figure 3*.

The panelists agreed that patients like telehealth, which offers benefits such as less travel time and lower costs. For clinicians, benefits included fewer no-show visits, greater ease of soliciting collateral information from caregivers, and the ability to observe patients in their own environment. However, the panelists agreed that telehealth may limit the ability to perform a thorough neurological examination. Technical issues, such as poor connection, video quality, and/or camera placement, can be obstacles for effectively diagnosing and managing TD. It can also be difficult to observe involuntary movements during a virtual visit because of segmented/obstructed views of the body or difficulty eliciting hyperkinetic movements. Furthermore, virtual assessment of other DIMDs which can coexist with TD (e.g., observing tone, gait and ancillary signs of parkinsonism) can also be more challenging.

### BEST PRACTICES FOR TD TELEHEALTH

The panel agreed that telehealth should not be considered a substitute for face-to-face visits for patients who are at risk of developing DIMDs such as TD, as lack of periodic in-person interaction may compromise the doctor–patient interaction and potentially lead to suboptimal diagnosis and treatment (*Fig. 3*). However, when used in combination with periodic in-person visits, telehealth can be part of an overall strategy for improving TD diagnosis and treatment. The panel recommended that patients have an in-person TD assessment within 6 months before the first virtual visit and at least one in-person assessment every 6 months thereafter.

Additional best practices included implementing video during all virtual visits and encouraging family members and/or caregivers to participate in the visit to help better understand the overall impact of involuntary movements on both patients and their care partners. The panel also recommended providing patients with clear instructions about the technology set-up before their telehealth visit, as detailed below.

**Fig. 4.**
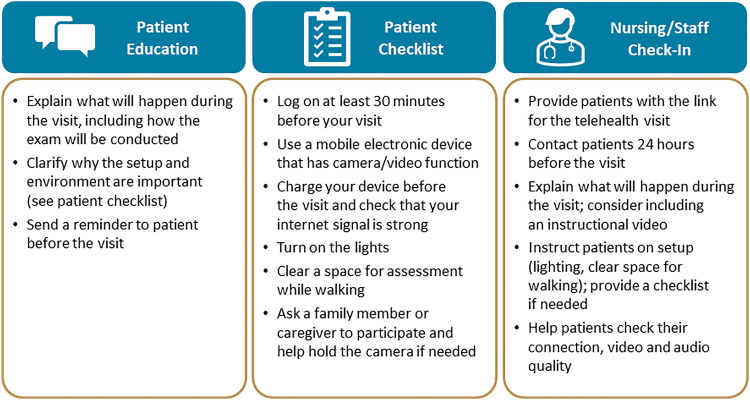
Recommendations: Preparing for the virtual appointment.

Specific recommendations to help patients prepare for their virtual appointments are presented in *Figure 4*. Patients should be educated about what will happen during the visit, including how examinations will be conducted and why the setup and environment are important. Providing a checklist can help patients prepare the technology and physical space for the virtual visit. In addition, nurses or other staff members should check in with the patient 24 h before the scheduled visit; during these check-ins, they can help patients test their technology and provide reminders about room and camera setup. If the patient is not able or comfortable conducting a virtual visit from their home, HCPs or other staff can offer an alternative examination location with appropriate technology, such as a telemedicine office or standardized telehealth room.

### OVERALL STRATEGIES FOR IMPROVING TD SCREENING, DIAGNOSIS, AND TREATMENT

**Fig. 5.**
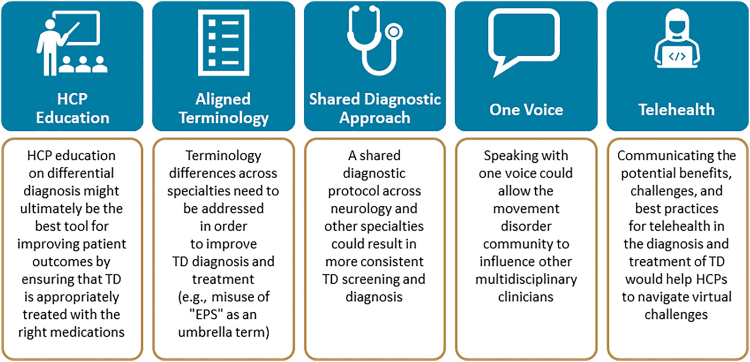
Overall strategies: improving TD screening, diagnosis, and treatment. EPS, extrapyramidal symptoms; HCP, health care provider; TD, tardive dyskinesia.

HCP education on the differential diagnosis of TD may be the best tool for improving patient outcomes, as this would ensure that TD is appropriately identified and treated with the right medications in both in-person and virtual settings (*Fig. 5*). In addition, alignment within the movement disorder community, particularly with regard to terminology and diagnostic protocols, is needed to help multidisciplinary clinicians (e.g., general practitioners) identify patients who have or are at risk for developing DIMDs. Finally, open communication among patients, caregivers, and HCPs about the benefits, challenges, and best practices of telehealth for TD can facilitate effective virtual diagnosis and treatment.

## Discussion

Despite a growing body of published literature on the incorporation of telehealth into the clinical care of patients with movement disorders such as Parkinson's disease, there is little published insight on its use in patients with DIMDs, including TD.^14,15^ Most studies have evaluated the feasibility of telehealth as a follow-up service to patients with an established diagnosis (usually Parkinson's disease), after an initial in-person evaluation with a detailed medical history and neurological examination.^14,15,32–36^ To date, there are only limited data on the use of telehealth in screening, diagnosing, and managing TD. This layered, qualitative study gathered information from TD experts on their experiences translating TD management (i.e., diagnosis, assessment, and treatment) to virtual care.

The expert panel agreed that telehealth can offer some advantages over traditional in-person visits: fewer no-show visits, better patient access (less time and travel costs), and easier for caregivers/families to participate and provide collateral information. Previous studies of telehealth in patients with movement disorders have shown increased patient satisfaction, reduced cost, and improved access for patients who have difficulty traveling or live in rural areas.^16–18,32,37^

Despite these advantages, the panelists noted that the inherent challenges in the identification and accurate diagnosis of movement disorders, including the differentiation of TD from other DIMDs, can be further complicated by the limitations of telehealth, such as segmented/obstructed views of the patient, inability to conduct a complete neurological examination, poor connection, communication difficulties, and limited access to appropriate technology.

In a recent observational survey study on telehealth during COVID-19, neurology and psychiatry specialists reported similar challenges in telehealth assessment of DIMDs, including difficulty assessing gait/balance, insufficient training for clinicians and staff, patients with limited access to computers and telephones, and greater difficulty obtaining reimbursements.^38^

Given these challenges, telehealth should not take the place of in-person visits for individuals who are at risk of TD; rather, it should be used as a helpful complement to clinical care. The panelists recommended that patients at risk for TD should have an in-person TD assessment within 6 months before the first virtual visit and at least one in-person assessment every 6 months thereafter, which aligns with the APA guidelines for TD screening and evaluation.^27^ The expert panel agreed on several key points for TD assessments with telehealth, including the importance of incorporating video in all telehealth visits, as audio-only interactions are insufficient to evaluate TD. To encourage patient participation during virtual visits, panelists recommended familiarizing patients with the telehealth process and technology before the appointment.

In a study of patient-physician communication during telehealth and in-person consultations, unfamiliarity with technology and a perceived loss of personal connection may have contributed to lower patient participation during telehealth versus in-person visits.^39^ Panelists also recommended engaging family and caregivers, as they can provide collateral information about the presence and impact of TD symptoms. In addition, family members can be a valuable partner in discussions about treatment options (e.g., timing of once-daily dosing [valbenazine] or twice-daily dosing with meals [deutetrabenazine]) and can help to improve treatment adherence.

Finally, the panel recommended continued HCP education on the clinical features of DIMDs. A useful resource to facilitate recognition and appropriate treatment of TD is a recent review by Hauser et al, which includes detailed descriptions of the different movements associated with TD and other DIMDs, with accompanying links to videos illustrating TD and non-TD movements.^22^

With proper training, TD screening can be done by any trained clinician (i.e., physician, nurses, allied health professionals).^40,41^ Screening could include informal evaluations, such as the simple-to-administer rating scales that were used in the real-world RE-KINECT study.^21^ These included clinician- and patient-rated severity of abnormal movements (“none,” “some,” or “a lot”) in four body regions (head/face, trunk/neck, upper extremities, lower extremities) and patient-rated impact (“none,” “some,” or “a lot”) of these abnormal movements in seven functional areas (continuing usual activites, talking, eating, breathing, being productive, taking care of self, and socializing). Although not tested in telehealth settings, it seems reasonable to expect that these scales would be appropriate for use during virtual visits or as part of a previsit patient questionnaire.

Some validated scales used to assess TD in person may also be adaptable to telehealth. Video rating for the AIMS is already common practice in TD clinical trials.^42–46^ A structured AIMS examination can be reliably performed over video telehealth with a full-body view,^47^ or a modified examination (6/7 regions) can be performed with an upper-body view. There is also room for innovation with virtual-friendly instruments, such as the recent MIND-TD questionnaire. MIND-TD guides clinicians through a short screen for abnormal movements, an interview about physical/functional difficulties, and visual observation with telehealth-appropriate activation maneuvers for a modified AIMS assessment.

## Conclusions

The future of psychiatric and neurological care may be moving toward hybrid models of care that balance in-office visits with virtual visits. To ensure that patients with TD receive appropriate care, future policy for telehealth should weigh the benefits and risks of audio-only telehealth, improve access to appropriate video technology during virtual visits (an estimated 45–50% of telehealth visits during the pandemic were audio-only),^48,49^ and safeguard continued access to and proper incentives for in-person visits. In addition, future studies should evaluate outcomes resulting from hybrid care delivery models. Finally, the development of new technologies, such as wearable devices or sensors, may enhance future virtual visits.

## References

[1] Institute of Medicine (US) Committee on Evaluating Clinical Applications of Telemedicine. Telemedicine: A Guide to Assessing Telecommunications in Health Care. (Field MJ. ed.). National Academies Press (US): Washington (DC); 1996. 20845554

[2] Tuckson RV, Edmunds M, Hodgkins ML. Telehealth. N Engl J Med 2017;377(16):1585–1592. 29045204 10.1056/NEJMsr1503323

[3] Koonin LM, Hoots B, Tsang CA, et al. Trends in the use of telehealth during the emergence of the COVID-19 pandemic—United States, January-March 2020. MMWR Morb Mortal Wkly Rep 2020;69(43):1595–1599. 33119561 10.15585/mmwr.mm6943a3 PMC7641006

[4] Chen JA, Chung WJ, Young SK, et al. COVID-19 and telepsychiatry: Early outpatient experiences and implications for the future. Gen Hosp Psychiatry 2020;66:89–95. 32750604 10.1016/j.genhosppsych.2020.07.002 PMC7347331

[5] Shachar C, Engel J, Elwyn G. Implications for telehealth in a postpandemic future: Regulatory and privacy issues. JAMA 2020;323(23):2375–2376. 32421170 10.1001/jama.2020.7943

[6] Weiner JP, Bandeian S, Hatef E, et al. In-person and telehealth ambulatory contacts and costs in a large US insured cohort before and during the COVID-19 pandemic. JAMA Netw Open 2021;4(3):e212618. 33755167 10.1001/jamanetworkopen.2021.2618 PMC7988360

[7] Patel SY, Mehrotra A, Huskamp HA, et al. Trends in outpatient care delivery and telemedicine during the COVID-19 pandemic in the US. JAMA Intern Med 2021;181(3):388–391. 10.1001/jamainternmed.2020.5928 PMC7670397 33196765

[8] Schulz T, Long K, Kanhutu K, et al. Telehealth during the coronavirus disease 2019 pandemic: Rapid expansion of telehealth outpatient use during a pandemic is possible if the programme is previously established. J Telemed Telecare 2020:1357633X20942045. 10.1177/1357633X20942045 PMC9124640 32686556

[9] American Psychiatric Association. Psychiatrists Use of Telepsychiatry During COVID-19 Public Health Emergency: Survey Results. 2021. Available from: https://www.psychiatry.org/File%20Library/Psychiatrists/Practice/Telepsychiatry/APA-Telehealth-Survey-2020.pdf [Last accessed: November 11, 2021 ].

[10] Trends Shaping the Health Economy: Telehealth. Trilliant Health Report. Available from: https://www.trillianthealth.com/insights/reports/telehealth-trends-shaping-the-health-economy [Last accessed: March 9, 2022].

[11] Telehealth is here to stay. Nat Med 2021;27(7):1121. 10.1038/s41591-021-01447-x 34267379

[12] Carpenter AB, Sheppard E, Atabaki S, et al. A symposium on the clinic of the future and telehealth: Highlights and future directions. Cureus 2021;13(5):e15234. 34178544 10.7759/cureus.15234 PMC8223952

[13] Centers for Medicare & Medicaid Services. Calendar Year (CY) 2022 Medicare Physician Fee Schedule Proposed Rule. 2021; Available from: https://www.cms.gov/newsroom/fact-sheets/calendar-year-cy-2022-medicare-physician-fee-schedule-proposed-rule [Last accessed: November 2, 2021].

[14] Mulroy E, Menozzi E, Lees AJ, et al. Telemedicine in movement disorders: Lecons du COVID-19. Mov Disord 2020;35(11):1893–1896. 32881108 10.1002/mds.28297

[15] Srinivasan R, Ben-Pazi H, Dekker M, et al. Telemedicine for hyperkinetic movement disorders. Tremor Other Hyperkinet Mov (N Y) 2020;10:10.7916/tohm.v0.698. 32195039 10.7916/tohm.v0.698 PMC7070700

[16] Wechsler LR. Advantages and limitations of teleneurology. JAMA Neurol 2015;72(3):349–354. 25580942 10.1001/jamaneurol.2014.3844

[17] Seritan AL, Heiry M, Iosif AM, et al. Telepsychiatry for patients with movement disorders: A feasibility and patient satisfaction study. J Clin Mov Disord 2019;6:1. 10.1186/s40734-019-0077-y PMC6555013 31183157

[18] Ben-Pazi H, Browne P, Chan P, et al. The promise of telemedicine for movement disorders: An interdisciplinary approach. Curr Neurol Neurosci Rep 2018;18(5):26. 29654523 10.1007/s11910-018-0834-6

[19] Bestsennyy OG, Gilbert G, Harris A, et al. Telehealth: A quarter-trillion-dollar post-COVID-19 reality? 2021. Available from: https://www.mckinsey.com/industries/healthcare-systems-and-services/our-insights/telehealth-a-quarter-trillion-dollar-post-covid-19-reality [Last accessed: November 2, 2021].

[20] Dorsey ER, Bloem BR, Okun MS. A new day: The role of telemedicine in reshaping care for persons with movement disorders. Mov Disord 2020;35(11):1897–1902. 32870517 10.1002/mds.28296

[21] Caroff SN, Yeomans K, Lenderking WR, et al. RE-KINECT: A prospective study of the presence and healthcare burden of tardive dyskinesia in clinical practice settings. J Clin Psychopharmacol 2020;40(3):259–268. 32332461 10.1097/JCP.0000000000001201 PMC7190052

[22] Hauser RA, Meyer JM, Factor SA, et al. Differentiating tardive dyskinesia: A video-based review of antipsychotic-induced movement disorders in clinical practice. CNS Spectr 2020:1–10. 10.1017/S109285292000200X PMC9249122 33213556

[23] Jain R, Correll CU. Tardive dyskinesia: Recognition, patient assessment, and differential diagnosis. J Clin Psychiatry 2018;79(2):nu17034ah1c. 10.4088/JCP.nu17034ah1c 29570969

[24] McEvoy J, Gandhi SK, Rizio AA, et al. Effect of tardive dyskinesia on quality of life in patients with bipolar disorder, major depressive disorder, and schizophrenia. Qual Life Res 2019;28(12):3303–3312. 31435866 10.1007/s11136-019-02269-8 PMC6863950

[25] Jackson R, Brams MN, Citrome L, et al. Assessment of the impact of tardive dyskinesia in clinical practice: Consensus panel recommendations. Neuropsychiatr Dis Treat 2021;17:1589–1597. 34079257 10.2147/NDT.S310605 PMC8164384

[26] Cutler AJ, Caroff SN, Tanner CM, et al. Caregiver-reported burden in RE-KINECT: Data From a prospective real-world tardive dyskinesia screening study. J Am Psychiatry Nurses Assoc. 2021. Available from: https://pubmed.ncbi.nlm.nih.gov/34154444/ [Last accessed: November 28, 2022]. 10.1177/10783903211023565 PMC10492432 34154444

[27] Keepers GA, Fochtmann LJ, Anzia JM, et al. The American Psychiatric Association Practice Guideline for the treatment of patients with schizophrenia. Am J Psychiatry 2020;177(9):868–872. 32867516 10.1176/appi.ajp.2020.177901

[28] Caroff SN, Citrome L, Meyer J, et al. A modified Delphi consensus study of the screening, diagnosis, and treatment of tardive dyskinesia. J Clin Psychiatry 2020;81(2):19cs12983. 10.4088/JCP.19cs12983 31995677

[29] Busetto L, Wick W, Gumbinger C. How to use and assess qualitative research methods. Neurol Res Pract 2020;2:14. 33324920 10.1186/s42466-020-00059-z PMC7650082

[30] Metaplan. Metaplan Basic Techniques: Moderating group discussions using the Metaplan approach. 2021. Available from: https://www.metaplan.com/wp-content/uploads/2021/04/Metaplan_Basiswissen_engl.pdf [Last accessed: October 18, 2021].

[31] Shah C, Lundt L, Vanderhoef D, et al. What's next for tardive dyskinesia? Expert insights from a cross-disciplinary virtual treatment panel. Ann Neurol 2021;90(suppl 27):S150.

[32] Dorsey ER, Venkataraman V, Grana MJ, et al. Randomized controlled clinical trial of “virtual house calls” for Parkinson disease. JAMA Neurol 2013;70(5):565–570. 23479138 10.1001/jamaneurol.2013.123 PMC3791511

[33] Barbour PJ, Arroyo J, High S, et al. Telehealth for patients with Parkinson's disease: Delivering efficient and sustainable long-term care. Hosp Pract (1995) 2016;44(2):92–97. 26982525 10.1080/21548331.2016.1166922

[34] Korn RE, Wagle Shukla A, Katz M, et al. Virtual visits for Parkinson disease: A multicenter noncontrolled cohort. Neurol Clin Pract 2017;7(4):283–295. 28840919 10.1212/CPJ.0000000000000371 PMC5566796

[35] Hanson RE, Truesdell M, Stebbins GT, et al. Telemedicine vs office visits in a movement disorders clinic: Comparative satisfaction of physicians and patients. Mov Disord Clin Pract 2019;6(1):65–69. 30746418 10.1002/mdc3.12703 PMC6335514

[36] Cubo E, Gabriel-Galan JM, Martinez JS, et al. Comparison of office-based versus home Web-based clinical assessments for Parkinson's disease. Mov Disord 2012;27(2):308–311. 22173694 10.1002/mds.24028

[37] Davis LE, Coleman J, Harnar J, et al. Teleneurology: Successful delivery of chronic neurologic care to 354 patients living remotely in a rural state. Telemed J E Health 2014;20(5):473–477. 24617919 10.1089/tmj.2013.0217

[38] Bera R, Franey E, Martello K, et al. TeleSCOPE: A real-world study of telehealth for the detection and treatment of drug-induced movement disorders. CNS Spectr 2022;27(2):250.

[39] Agha Z, Roter DL, Schapira RM. An evaluation of patient-physician communication style during telemedicine consultations. J Med Internet Res 2009;11(3):e36. 19793720 10.2196/jmir.1193 PMC2802255

[40] Kane JM, Correll CU, Nierenberg AA, et al. on behalf of the Tardive Dyskinesia Assessment Working Group. Revisiting the Abnormal Involuntary Movement Scale: Proceedings from the Tardive Dyskinesia Workshop. J Clin Psychiatry 2018;79(3):17cs11959. 10.4088/JCP.17cs11959 29742330

[41] Bark N, Florida D, Gera N, et al. Evaluation of the routine clinical use of the Brief Psychiatric Rating Scale (BPRS) and the Abnormal Involuntary Movement Scale (AIMS). J Psychiatr Pract 2011;17(4):300–303. 21775833 10.1097/01.pra.0000400269.68160.e6

[42] Hauser RA, Factor SA, Marder SR, et al. KINECT 3: A phase 3 randomized, double-blind, placebo-controlled trial of valbenazine for tardive dyskinesia. Am J Psychiatry 2017;174(5):476–484. 28320223 10.1176/appi.ajp.2017.16091037

[43] Factor SA, Remington G, Comella CL, et al. The effects of valbenazine in participants with tardive dyskinesia: Results of the 1-Year KINECT 3 extension study. J Clin Psychiatry 2017;78(9):1344–1350. 29141124 10.4088/JCP.17m11777

[44] O'Brien CF, Jimenez R, Hauser RA, et al. NBI-98854, a selective monoamine transport inhibitor for the treatment of tardive dyskinesia: A randomized, double-blind, placebo-controlled study. Mov Disord 2015;30(12):1681–1687. 26346941 10.1002/mds.26330 PMC5049616

[45] Anderson KE, Stamler D, Davis MD, et al. Deutetrabenazine for treatment of involuntary movements in patients with tardive dyskinesia (AIM-TD): A double-blind, randomised, placebo-controlled, phase 3 trial. Lancet Psychiatry 2017;4(8):595–604. 28668671 10.1016/S2215-0366(17)30236-5

[46] Fernandez HH, Factor SA, Hauser RA, et al. Randomized controlled trial of deutetrabenazine for tardive dyskinesia: The ARM-TD study. Neurology 2017;88(21):2003–2010. 28446646 10.1212/WNL.0000000000003960 PMC5440239

[47] Amarendran V, George A, Gersappe V, et al. The reliability of telepsychiatry for a neuropsychiatric assessment. Telemed J E Health 2011;17(3):223–225. 21443440 10.1089/tmj.2010.0144

[48] Chen J, Li KY, Andino J, et al. Predictors of audio-only versus video telehealth visits during the COVID-19 pandemic. J Gen Intern Med 2022;37(5):1138–1144. 34791589 10.1007/s11606-021-07172-y PMC8597874

[49] Benjenk I, Franzini L, Roby D, et al. Disparities in audio-only telemedicine use among Medicare beneficiaries during the coronavirus disease 2019 pandemic. Med Care 2021;59(11):1014–1022. 34534186 10.1097/MLR.0000000000001631 PMC8516710

